# A Retrospective Study of Demographic, Socio-Economic and Healthcare Access Disparities Among Patients With Depression in the USA

**DOI:** 10.7759/cureus.86781

**Published:** 2025-06-26

**Authors:** Azka Iqbal, Richa Rajendrakumar Patel, Kawtar Haimeur, Geetha Aanagouni, Isha Samhitha Purama

**Affiliations:** 1 Internal Medicine, St. George's University, St. George's, GRD; 2 Internal Medicine, Baroda Medical College, Vadodara, IND; 3 General Medicine, Mohammed VI University of Health and Sciences, Casablanca, MAR; 4 Internal Medicine, MediCiti Institute of Medical Sciences, Medchal, IND; 5 Internal Medicine, Chalmeda Anand Rao Institute of Medical Sciences, Karimnagar, IND

**Keywords:** behavioural risk factor surveillance system, demographic analysis, depression, healthcare access, insurance coverage, mental health, odds ratio, public health policy, socio-economic status

## Abstract

Introduction

Depression is a multifactorial psychological condition influenced by a complex interplay of demographic, socioeconomic, and healthcare access factors. Understanding these associations is essential to develop effective, inclusive, and targeted mental health interventions. Globally, and particularly in the United States, disparities in mental healthcare organization and access contribute to unequal outcomes.

Methodology

This cross-sectional observational study utilized the 2021 Behavioral Risk Factor Surveillance System (BRFSS) dataset comprising 438,693 respondents across the United States. After applying exclusion criteria, 85,398 participants were included in the final analysis. Depression was the dependent variable, and independent variables included age, gender, race, education, employment, income, mental/physical health status, insurance type, and access to healthcare providers. Binomial logistic regression was used to calculate odds ratios (ORs), 95% confidence intervals (CIs), and p-values.

Results

Participants reporting 14 or more days of poor mental health had a significantly higher likelihood of depression (OR = 12.41; 95% CI: 11.77-13.08; *p* < 0.0001). Those with multiple physical health issues (OR = 1.56; 95% CI: 1.49-1.64; *p* < 0.0001) and more than one healthcare provider (OR = 1.21; 95% CI: 1.16-1.26; *p* < 0.0001) also had higher odds. Insurance types like Medicaid (OR = 1.83; 95% CI: 1.71-1.95; *p* < 0.0001) and military-related coverage (OR = 1.68; 95% CI: 1.54-1.85; *p* < 0.0001) were strongly associated with depression. Conversely, those who did not face medication cost-related issues had a lower likelihood of depression (OR = 0.62; 95% CI: 0.58-0.66; *p* < 0.0001).

Conclusion

Significant associations exist between depression and various demographic, socioeconomic, health, and healthcare access factors. These findings underscore the need for systemic reforms in mental healthcare delivery and support the development of targeted policies to reduce disparities and improve mental health outcomes across diverse populations.

## Introduction

Depressive disorder, a prevalent mental health condition affecting approximately 3.8% of the global population, poses significant challenges worldwide [1]. Despite its widespread impact, depression remains undertreated, with around half of affected adults lacking proper diagnosis and treatment due to various barriers, including limited access to mental healthcare services, lack of awareness, and affordability concerns [2]. Financial insecurity can lead to chronic stress, reduced access to quality healthcare, and limited ability to meet basic needs, all of which contribute to poor mental health outcomes. Similarly, employment status plays a critical role, with unemployed individuals or those in unstable jobs facing higher risks of depression due to factors such as financial strain, loss of identity, and social isolation. These socioeconomic factors not only affect the onset and course of depression but also influence help-seeking behavior and treatment outcomes. The disparities in mental healthcare access and utilization are particularly pronounced in the United States, where systematic healthcare practices contribute to unequal distribution of services [3, 4].

Given the complexity of depression and its multifactorial determinants, understanding the interplay between demographic, socioeconomic, and healthcare access factors is crucial for informing effective intervention strategies. The Behavioral Risk Factor Surveillance System (BRFSS), a comprehensive health-related telephone survey conducted across the United States, provides a valuable resource for investigating these relationships [5-7].

This study aims to evaluate the associations among demographic, socioeconomic, health status, and healthcare access factors in individuals suffering from depression in the USA and to provide valuable insights into the multifaceted nature of depression, thus contributing to the development of targeted interventions and evidence-based policies for promoting mental health and well-being in diverse populations.

## Materials and methods

Study design and data source

This observational cross-sectional study was conducted using data from the 2021 Behavioral Risk Factor Surveillance System (BRFSS), a publicly available and nationally representative telephone-based survey managed by the Centers for Disease Control and Prevention (CDC). Data extraction was performed on January 17, 2024.

Study population

The initial BRFSS dataset included 438,693 participants. After applying exclusion criteria, 85,398 participants were included in the final analysis.

Exclusion criteria

Participants were excluded if they had incomplete or missing responses for the primary outcome (depression) or key independent variables. Additionally, individuals who opted out of modules relevant to this study were excluded.

Variables

Dependent Variable

Self-reported depression, assessed by whether respondents had ever been told they had a depressive disorder including depression, major depression, dysthymia, or minor depression (variable: ADDEPEV3).

Independent Variables

These included demographic factors (age group, gender, race/ethnicity), socioeconomic factors (education level, employment status, annual household income), health status indicators (number of days mental or physical health was not good), healthcare access (presence of a personal healthcare provider, type of insurance coverage), and affordability of care (whether the respondent needed to see a doctor in the past 12 months but could not due to cost).

Age was categorized into six standard BRFSS age groups. Race was classified into eight BRFSS-defined race/ethnicity categories. Education and income were grouped according to BRFSS standard cutoffs. Health status variables included _MENT14D (mental health) and _PHYS14D (physical health). Access to care was measured using responses to PERSDOC3 (personal doctor) and PRIMINSR (insurance type). Affordability of care was assessed via MEDCOST1.

All abbreviations used above follow BRFSS-derived variable names and have been defined on first use.

Data analysis

Descriptive statistics were used to report frequencies and percentages for all categorical variables. All analyses were conducted using the Web Enabled Analysis Tool (WEAT) provided by the CDC.

To examine associations between depression and independent variables, binomial logistic regression was performed. The analysis produced odds ratios (ORs), 95% confidence intervals (CIs), and p-values. A p-value of <0.05 was considered statistically significant.

Data management was performed using Microsoft Excel (Microsoft Corp., Redmond, WA, USA), and graphical analyses were carried out using GraphPad Prism version 9.4.1 (GraphPad Software, San Diego, CA, USA).

## Results

In this study, a total of 438,693 participants were initially included in the BRFSS dataset for the selected year and location. During the study, some participants were excluded based on specific criteria, resulting in a final sample size of 85,398 participants for the analysis. The exclusions were made due to various reasons, which are detailed in the subsequent paragraph*.*

Demographic and socio-economic characteristics of the study population are presented in Table 1. Most participants were female (n=56,895; 64.2%), and belonged to the 25-34 years age group (11,666; 20.2%). The racial composition of the population included 70.3% (n=67,757) White, 9.3% (n=4970) Black, 13.7% (n=6681) Hispanic, and smaller percentages for other racial categories. Education levels varied, with 35.1% (n=26,635) having completed Grade 12 or GED (High school graduate), and 25% (n=31,021) having completed four years or more of college. Employment status and annual household income were also diverse among the participants.

**Table 1:** Demographic and socio-economic characteristics of the study population. Values are represented as N (%).

Variable	No. (%)
Demographic	(n=85,398)
Age	
Age 18 to 24	6510 (15.3)
Age 25 to 34	11,666 (20.2)
Age 35 to 44	13,197 (16.8)
Age 45 to 54	13,909 (15)
Age 55 to 64	17,190 (16.2)
Age 65 or older	22,926 (16.5)
Gender	
Male	28,503 (35.8)
Female	56,895 (64.2)
Race	
White, non-Hispanic	67,757 (70.3)
Black, non-Hispanic	4,970 (9.3)
Hispanic	6,681 (13.7)
American Indian/Alaskan Native, non-Hispanic	1,448 (1.1)
Asian, non-Hispanic	995 (2.9)
Native-Hawaiian/other Pacific Islander, non-Hispanic	212 (0.1)
Other race, non-Hispanic	954 (0.8)
Multiracial, non-Hispanic	2,381 (1.9)
Socio-economic	
Education level	(n=85,166)
Never attended school or only kindergarten	106 (0.3)
Grades 1-8 (Elementary)	1,511 (3.4)
Grades 9-11 (Some high school)	4,313 (9.2)
Grade 12 or GED (High school graduate)	21,580 (35.1)
College 1 year to 3 years (Some college or technical)	26,635 (35.1)
College 4 years or more (College graduate)	31,021 (25)
Employment status	(n=84,270)
Employed for wages	33,358 (42.5)
Self-employed	5,400 (6.7)
Out of work for 1 year or more	3,542 (5.1)
Out of work for less than 1 year	2,801 (4.2)
A homemaker	3,844 (5.6)
A student	2,598 (6)
Retired	20,005 (15.4)
Unable to work	12,722 (14.6)
Annual household income	(n=70,106)
Income < $10,000	3928 (6.3)
$10,000 <= Income < $15,000	4126 (5.5)
$15,000 <= Income < $20,000	4432 (6)
$20,000 <= Income < $25,000	5599 (7.7)
$25,000 <= Income < $35,000	10382 (14.7)
$35,000 <= Income < $50,000	9833 (13.2)
$50,000 <= Income < $75,000	11,080 (11.5)
$75,000 <= Income < $100,000	8,000 (11.5)
$100,000 <= Income < $150,000	7,664 (11.3)
$150,000 <= Income < $200,000	2,788 (4.4)
Income >= $200,000	2,274 (4.1)

Table 2 outlines the health status and healthcare access of the study population. Mental health, physical health, healthcare provider, and insurance status were assessed. Most participants reported having good mental health on most days (22.4%), while the distribution of physical health was balanced among those with one or more health issues. The study population had varied healthcare provider arrangements, and the majority were covered by a plan purchased through an employer/union (37.3%). About 17.7% (n=12,223) of participants reported needing to see a doctor in the past 12 months but could not afford it*.*

**Table 2:** Health status & healthcare access of study population. REF: Reference value

Variable	No. (%)
Health Status	
Mental Health	(n=83,281)
0 days when mental health not good (REF)	21,532 (22.4)
1-13 days when mental health not good	30,089 (35.7)
14+ days when mental health not good	31,660 (41.9)
Physical Health	(n=83,195)
Yes, only one (REF)	38,780 (46.6)
More than one	24,862 (31.3)
No	19,553 (22.1)
Healthcare provider	(n=84,875)
Yes, only one (REF)	47,490 (54.3)
More than one	29,311 (32.8)
No	8,074 (13)
Insurance	(n=82,644)
A plan purchased through employer/union (REF)	28,407 (37.3)
A private plan bought on your own	5997 (8.3)
Medicare	23,974 (20.9)
Medigap	106 (0.1)
Medicaid	10028 (13.1)
Children's Health Insurance Program (CHIP)	38 (0.1)
Military-related health care (TRICARE/VA/CHAMP-VA)	3713 (4.4)
Indian Health Service	278 (0.2)
State-sponsored health plan	3158 (4.5)
Other government programs	2502 (3.4)
No coverage of any type	4443 (7.5)
Past 12 months, needed to see a doctor but could not afford (MEDCOST1)	(n=85,097)
Yes (REF)	12,223 (17.7)
No	72,874 (82.3)

**Figure 1:**
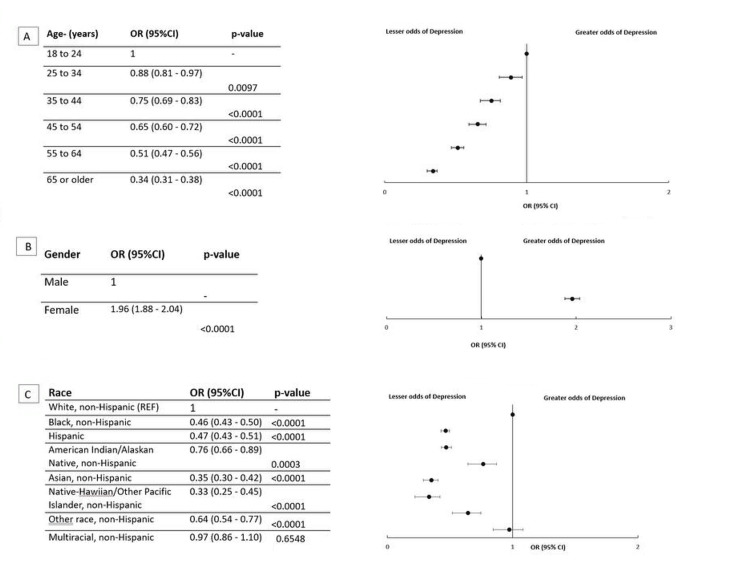
Odds ratios (OR) of depression calculated for age, gender, and race Figure 1A: OR for Age Figure 1B: OR for Gender Figure 1C: OR for Race p-value <0.05 was considered statistically significant.

**Figure 2:**
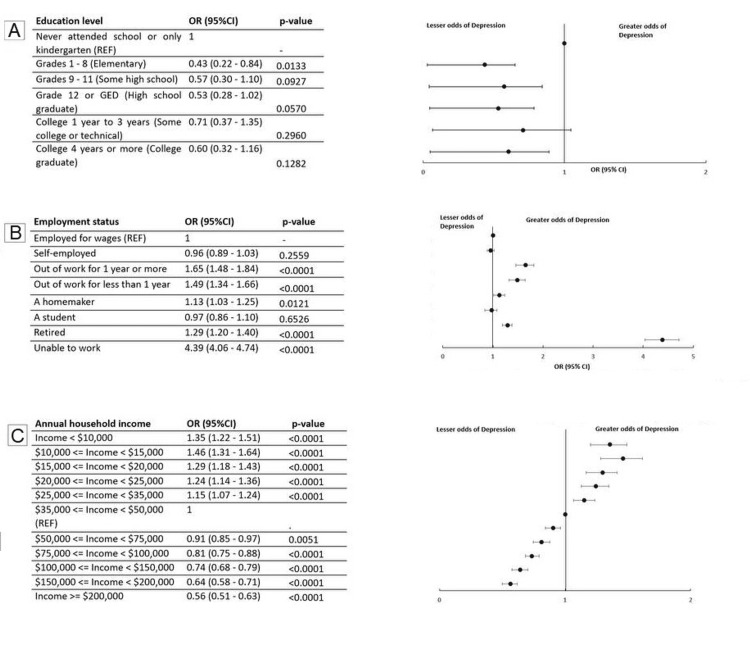
Odds ratios of depression calculated for education level, employment status and annual income Figure 2A: OR for Education Level Figure 2B: OR for Employment Status Figure 2C: OR for Annual Income p-value < 0.05 was considered statistically significant.

We investigated the association between various variables and mental health, MEDCOST1, insurance status, healthcare provider, physical health, gender, annual household income, employment status, education level, and race (Figures 1, 2). The investigation focused on the odds ratios (OR) with 95% confidence intervals (CI) and corresponding p-values*.*

Age

The analysis revealed that participants aged 25-34 years had the highest odds of reporting depression compared to the reference group aged 65 years or older. Younger adults, particularly those between 18 and 44 years, consistently showed elevated odds, suggesting increased vulnerability to mental health challenges during early adulthood. This trend aligns with existing literature, which attributes higher depression rates in this age group to transitional life stages, career pressures, and changing social dynamics.

Gender

Female participants demonstrated nearly double the odds of experiencing depression compared to male participants (OR = 1.96; 95% CI: 1.88-2.04; p < 0.0001). This significant disparity is well-documented in psychiatric epidemiology and may be influenced by hormonal, psychosocial, and cultural factors that increase the risk of depression among women.

Race/Ethnicity

White, non-Hispanic individuals had higher odds of depression compared to other racial and ethnic groups. While this finding is consistent with previous national reports, it is important to consider that underreporting, stigma, or limited healthcare access may influence lower reported rates of depression in some minority groups. Additionally, cultural resilience factors could also contribute to these observed differences.

Education

Individuals who had completed some college or technical education had higher odds of depression compared to both those with lower (high school or less) and higher (college graduate) education. This U-shaped association suggests that individuals with mid-level educational attainment may face unique stressors such as educational debt or job market instability, which could contribute to increased mental health burden.

Employment

Depression was strongly associated with employment status. Those who were unemployed - either currently or for more than a year - and those unable to work due to disability had significantly higher odds of depression. In contrast, employed individuals, students, and retirees had comparatively lower odds. This underscores the importance of stable employment and its protective role in mental well-being, as unemployment is often linked to financial insecurity and social isolation.

Income

An interesting pattern emerged regarding income. Participants in the $25,000-$35,000 bracket had the highest odds of depression, even more so than those in the lowest income group. Furthermore, individuals earning between $50,000 and $150,000 also showed elevated odds. These findings challenge the conventional assumption that higher income is always protective and suggest that individuals in middle-income groups may face stress related to rising costs of living, job demands, and maintaining social status.

Variables related to mental health

**Figure 3:**
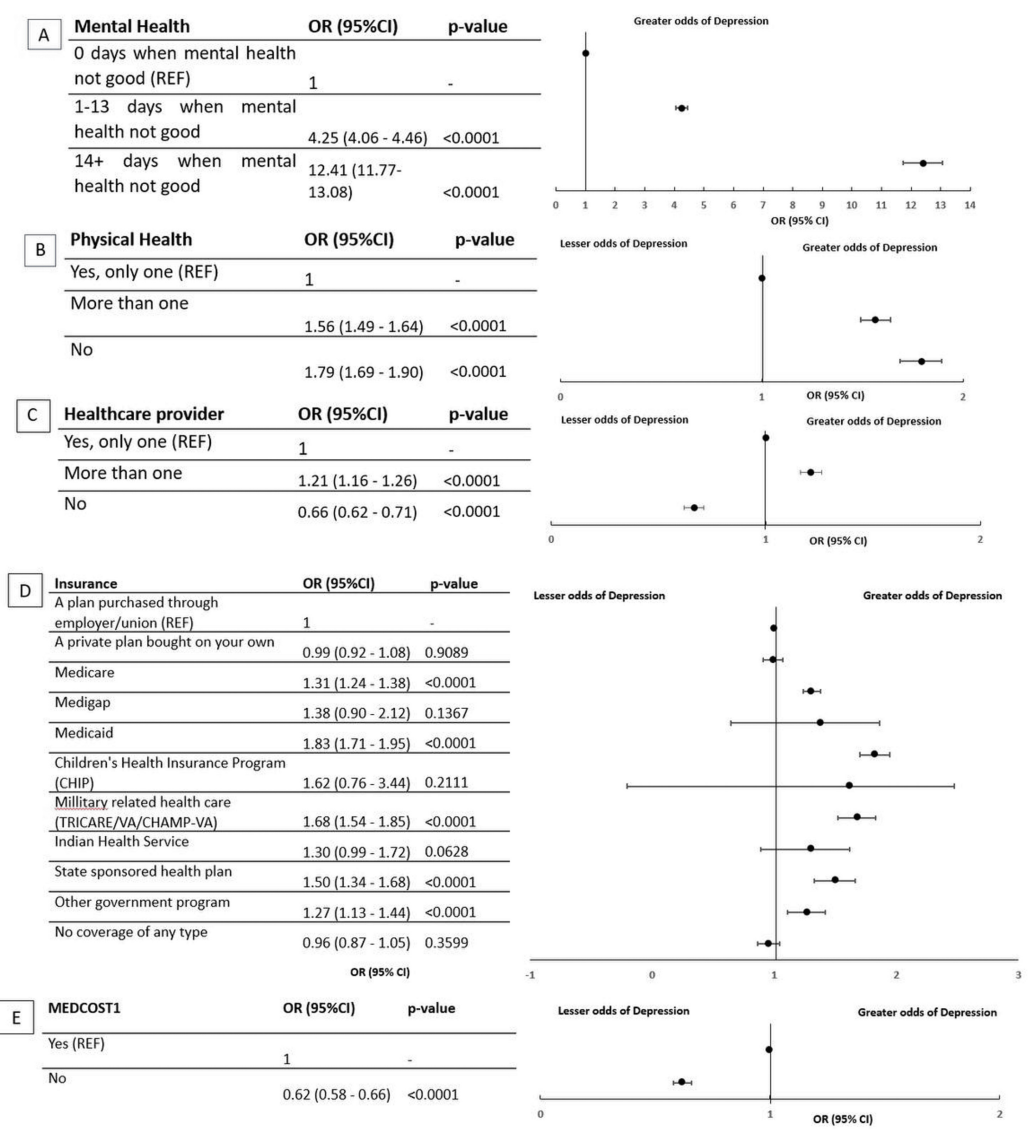
Variables for mental health Figure 3A: Mental health characteristics Figure 3B: Physical health characteristics Figure 3C: Healthcare provider characteristics Figure 3D: Insurance Figure 3E: MEDCOST1 (Indicator of whether the participant was unable to see a doctor in the past 12 months due to cost.) p-values <0.05 were considered statistically significant.

The investigation into mental health revealed a significant correlation with the number of days when mental health was not good. Participants reporting mental health issues for 1-13 days had an OR of 4.25 (95% CI: 4.06-4.46, p < 0.0001), while those with mental health issues for 14 or more days had a higher OR of 12.41 (95% CI: 11.77-13.08, p < 0.0001). This indicates a strong positive association between the duration of mental health issues and the likelihood of overall mental health problems (Figure 3).

Association with MEDCOST1

Participants without medical costs (MEDCOST1) demonstrated a lower likelihood of mental health issues, with an OR of 0.62 (95% CI: 0.58-0.66, p < 0.0001), suggesting a protective effect against mental health problems associated with lower medical costs.

Insurance status

Various types of insurance were examined for their association with mental health. Notably, Medicaid (OR: 1.83, 95% CI: 1.71-1.95, p < 0.0001), military-related healthcare (OR: 1.68, 95% CI: 1.54-1.85, p < 0.0001), state-sponsored health plans (OR: 1.50, 95% CI: 1.34-1.68, p < 0.0001), and other government programs (OR: 1.27, 95% CI: 1.13-1.44, p < 0.0001) were associated with an increased likelihood of mental health issues. Conversely, having no coverage of any type showed no significant association (OR: 0.96, 95% CI: 0.87-1.05, p = 0.3599).

Healthcare provider and physical health

Interestingly, participants who reported having no healthcare provider had lower odds of reported depression (OR = 0.66; 95% CI: 0.62-0.71; p < 0.0001) compared to those with a single provider. While this initially appears protective, it likely reflects underdiagnosis or reduced interaction with the healthcare system, rather than a true lower prevalence of depression. Individuals without regular care may have fewer opportunities for screening and diagnosis, suggesting the need to interpret this finding with caution.

## Discussion

The results of this study demonstrated associations between depression, defined as a mental health disorder characterized by persistent feelings of sadness, hopelessness, and a lack of interest or pleasure in daily activities, and other parameters like socioeconomic, demographic, health status and healthcare access.

Exploration of age-specific patterns within the context of depression prevalence has uncovered significant variations, notably highlighting that young adults, specifically those aged 25 to 34, exhibit the highest likelihood of reporting depressive symptoms. This aligns seamlessly with emerging evidence suggesting that the prevalence of anxiety disorders is intricately linked to youthfulness and female sex, a correlation corroborated by the findings of this investigation. The study underscores the observed trend wherein females surpass males in reporting depression [8].

Moreover, an examination of racial disparities reveals that individuals identifying as White, non-Hispanic, exhibit the highest rates of depression within the studied population. This corroborates existing literature which consistently supports the notion that individuals of White ethnicity are more predisposed to experiencing depression [9, 10].

Studies show that a few variations in mental healthcare experiences are due to patient preferences and cultural beliefs [4]. Most of the healthcare disparities (defined as difference in accessibility and quality between different demographics, commonly seen in the United States) are because of systematic, modifiable healthcare practices like doctor-patient communication, physician race and treatment preferences, delay in treatment, lack of pro-active health-care engagement and follow-up by patients particularly ethnic minorities in the USA [3, 8, 9-12]. Studies indicate a slight incidence of lower acculturation and even poorer health-service utilization by Hispanics.

A study performed among adults who experienced current depression or frequent mental distress titled “Alternatives in Assessing Mental Health-Care Disparities using Behavioral Risk Factor Surveillance System (BRFSS)” in 2018, demonstrates how gender, age, race, ethnicity, marital status, employment status, presence or absence of healthcare coverage are the risk factors and form the basis of demographic, socioeconomic and healthcare disparities in the United States [2-4, 12-17].

Interestingly, individuals who had attained high school graduation or had some college or technical education reported the highest prevalence of depressive symptoms. This observation stands in apparent contrast to existing literature, which commonly states that higher educational attainment correlates with improved mental health [18, 19]. The discrepancy observed in our study underscores the nuanced nature of the relationship between education and mental health, challenging conventional expectations. While some studies suggest a positive association between higher education levels and mental well-being, our findings suggest that certain subgroups, specifically those with intermediate educational achievements, may face distinct challenges or stressors influencing their mental health adversely.

Income disparities emerge as pivotal determinants in shaping mental health outcomes, as delineated by the findings of our study. Notably, the group with an income range of $25,000 to $35,000 reported the highest prevalence of depression, presenting a noteworthy departure from conventional expectations. Intriguingly, the cohorts with incomes between $50,000 to $75,000, $75,000 to $100,000, and $100,000 to $150,000 reported elevated levels of depression compared to the lowest income groups. This pattern aligns with previous research suggesting that higher income levels may indeed be associated with increased depression. The observed higher depression rates among the middle-income brackets prompt a critical examination of potential contributing factors [19]. The notion that the middle class may face unique challenges, potentially related to increased financial responsibilities, societal expectations, or economic pressures, emerges as a plausible explanation.

The observation that a significant proportion (41.9%) of the study population reported experiencing mental health challenges for 14 or more days is noteworthy. The group reporting more than one physical health issue constitutes 31.3% of the population. This finding underscores the complexity of health conditions experienced by individuals in this study and is supported by other studies [20]. The multifaceted nature of health issues necessitates comprehensive and holistic healthcare strategies that consider the coexistence of multiple conditions, potentially requiring integrated care approaches. Individuals with more than one healthcare provider account for 32.8% of the population. This signifies a substantial portion of the cohort seeking care from multiple sources.

The category with a plan purchased through an employer or union is the most prevalent, encompassing 37.3% of the population. This underscores the significant role of employer-sponsored insurance in providing coverage for a substantial proportion of the study participants. Ensuring the accessibility and affordability of such plans can be critical for promoting comprehensive healthcare coverage. The majority of the population (82.3%) did not report facing affordability challenges when needing to see a doctor in the past 12 months. This positive trend suggests that a significant portion of the study participants had financial access to necessary medical care. Other studies have confirmed these findings [21].

Our study and other studies reveal significant associations, such as the prevalence of mental health challenges among young adults, income-related disparities, and the impact of education and employment on mental health outcomes [22, 23]. To address these disparities, raising public awareness is crucial for targeted health policy initiatives. Physicians can play a pivotal role by implementing local initiatives, such as teleconsultation services for remote accessibility, and examining their practices to identify and address access gaps [24, 25]. Engaging in outreach interventions, particularly in schools and remote areas, allows physicians to educate communities and empower individuals. Collaborating with state advocacy groups provides an avenue to address root causes collectively and contribute to implementable solutions. This comprehensive approach empowers physicians to actively participate in reducing health care access disparities and fostering a more equitable healthcare landscape [26].

Limitations

We acknowledge that this study had limitations. Telephone surveys were employed instead of in-person interviews. Individuals lacking access to landlines or not using landlines or not at home were not included in the survey. The survey was conducted in English and various other languages. Individuals not proficient in these languages may represent underrepresented groups and were consequently omitted. The data relied on self-reports rather than being derived from clinic histories and examinations conducted in person. The data is presented in a cohort format rather than as individual data, leading to the inability to eliminate certain confounding factors. The survey questions varied annually and from state to state. Some combinations of race and ethnicity may be excluded from the analysis. The survey is cross-sectional and nationwide, limiting the ability to establish causation. Only associations can be identified.

## Conclusions

The study demonstrates associations between depression and various parameters, including socioeconomic, demographic, health status, and healthcare access. Further studies should be completed to address the interplay of these factors; future efforts should focus on implementing targeted interventions informed by a comprehensive understanding of the identified associations. By prioritizing initiatives that foster inclusivity, reduce disparities, and improve the overall accessibility and affordability of mental health services, we can contribute to creating a more equitable healthcare landscape for diverse populations.
